# External fixation is more suitable for intra-articular fractures of the distal radius in elderly patients

**DOI:** 10.1038/boneres.2016.17

**Published:** 2016-06-21

**Authors:** Chuang Ma, Qiang Deng, Hongwei Pu, Xinchun Cheng, Yuhua Kan, Jing Yang, Aihemaitijiang Yusufu, Li Cao

**Affiliations:** 1 Department of Orthopedics Center, First Affiliated Hospital of Xinjiang Medical University, Urumqi, China; 2 Department of Science and Research Education Center, First Affiliated Hospital of Xinjiang Medical University, Urumqi, China; 3 Carders Health Care NO. 4 Department of Xinjiang Uygur Autonomous Region People's Hospital, Urumqi, China; 4 Department of Orthopedics of the Central Hospital of Karamay, Karamay, China; 5 Department of Orthopedics Center, Fifth Affiliated Hospital of Xinjiang Medical University, Urumqi, China

## Abstract

The purpose of this study was to compare the functional outcomes, psychological impact, and complication rates associated with external fixation and volar or dorsal plating in relation to the functional parameters following treatment of intra-articular fractures of the distal radius (IFDR) in patients older than 65 years. We hypothesized that using volar or dorsal plating would improve functional outcomes, but that it would be associated with more complications and equivalent functional outcomes when compared with the external fixation group. A total of 123 consecutive patients suffering from IFDR were recruited into the study. The patients were measured for clinical, radiological, and psychosocial functioning outcomes and were followed up after 1 week and 3, 6 and 12 months. After 3 months, the plating group had better pronation (*P*=0.001), supination, (*P*=0.047) and extension (*P*=0.043) scores. These differences were somewhat attenuated by 6 months and disappeared at 1 year. The plating group had a greater occurrence of wound infection (*P*=0.043), tendonitis, (*P*=0.024) and additional surgery compared with the external fixation group. The only TNO-AZL Adult Quality of Life scores in the plating group that were lower than those in the external fixation group were in the “gross motor” category (walking upstairs, bending over, walking 500 yards*; P*=0.023). Internal fixation was more advantageous than external fixation in the early rehabilitation period; after 1 year the outcomes were similar. The plating group showed significantly higher levels of wound infection and tendonitis and had a greater need for additional surgeries.

## Introduction

Intra-articular fractures of the distal radius (IFDR) represent high energy, complex, unstable injuries, and account for approximately one-sixth of the fractures observed in emergency rooms.^1,2^ The risk of a poor outcome after IFDR increases with malunion and joint stiffness, and operations are often required to maintain a satisfactory anatomical position.^3^ Over the past 30 years, the surgical treatment of distal radius fractures has shifted from the use of cast immobilization to numerous surgical options, such as the use of external fixation and locking plates.^4,5^ Although many reports on the functional outcomes, psychological impacts and complication rates have been reported for the use of locked plating fixation or external fixation around the wrist,^6,7^ little has been written about these issues in elder patients.^8^ Chung *et al.* ^9^ reported complication rates after volar-locked plating of distal radius fractures; however, they included both young adults and adults older than 60 years in their analysis and did not focus on the elderly population.

Procedures for treating IFDR can be psychologically stressful because of their long duration and the frequency of complications, including soft tissue problems and restricted joint motion. Thus, the procedure used for treating IFDR can have a strong psychological impact on the patient.

There are obvious benefits and disadvantages of these two surgical techniques and the associated post-operative rehabilitation protocols. Some authors have previously compared external fixation with internal fixation, but there is insufficient evidence regarding which procedure has the best outcome in elderly patients.^10–13^

The primary purpose of this study was to compare the functional outcomes, psychological impacts, and complication rates in elderly patients whose IFDR was treated by either external or internal fixation.

## Materials and Methods

All studies involving human participants were approved by the First Affiliated Hospital of Xinjiang Medical University. Before operation, written informed consent was provided by participants for their clinical records to be used in this study, and the consent process was carried out by the treating surgeon. This case–control study excluded patients with open fractures, previous surgery involving the distal radius, concurrent injury involving the ipsilateral upper limb, and wrist pain predating this injury. Two hundred consecutive elderly patients suffering from IFDR between June 2009 and May 2015 were considered for the study. Of these, 123 patients met the inclusion criteria of a fracture of the distal radius requiring operative repair amenable to either open reduction and internal fixation or external fixation. The Institution Review Board approved the study, and the patients provided informed consent. Baseline clinical details, including the mechanisms of the fractures, and wrist, shoulder, and hand functional scores, were obtained. For each patient, a thorough and detailed patient history was obtained and a detailed physical examination was performed, with attention to neurovascular injury and deficits. The fractures were classified according to the system of the Orthopaedic Trauma Association (AO/OTA).^14^

The two groups were compared with respect to functional, clinical, and radiographic outcomes. Standard radiographs were obtained at presentation, including anteroposterior and lateral views. Radiographs of all displaced fractures were obtained. Measurements of radial inclination, height, tilt, and ulnar variance were made on post-reduction radiographs (Table 1).

There were no differences between the two groups in regard to age, gender, pattern of fracture, hand dominance, or number of pre-existing medical conditions (cardiovascular, endocrine, pulmonary, or kidney disease; Table 2). The patients were randomized into two groups according to whether they underwent internal fixation or external fixation. A number for each patient was placed in a sealed envelope by a blinded assistant and given to the treating surgeon. The envelopes were opened on the day of surgery, immediately before the operation. Fifty-eight patients were randomly assigned to the external fixation treatment group and 65 to the internal fixation treatment group. The criteria used to identify unacceptable reductions were a radial height shortening of >5 mm, radial inclination on a postero-anterior film <15°, intra-articular step off >2 mm, dorsal tilt >15° and volar tilt >20°.^15^

### Operative technique

All operations were performed by one of four senior orthopedic surgeons, who had been in practice for 6–10 years and had extensive experience performing external fixation or locked plating treatments. A full-time physiotherapist performed the pre- and post-operation clinical examinations. All the operations were performed under brachial plexus anesthesia or general anesthesia.

### External fixation

**Figure 1:**
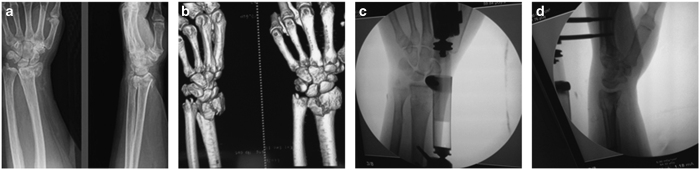
Radiographs of a 68-year-old man with a displaced intra-articular fracture of the distal radius that was treated by external fixation. (**a** and **b**) Anteroposterior and lateral radiograph before operation; (**c** and **d**) Anteroposterior and lateral radiograph during operation.

**Figure 2:**
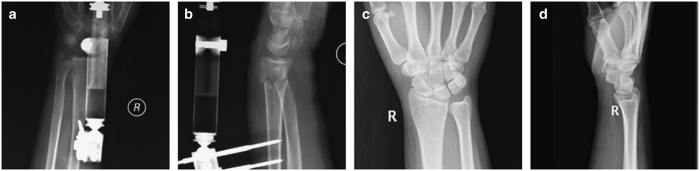
Radiographs of the distal radius with a comminuted intra-articular fracture after external fixation. (**a** and **b**) Anteroposterior and lateral radiographs after one month, (**c** and **d**) Anteroposterior and lateral radiographs after one year.

The external fixation group underwent closed reduction or open reduction with the placement of two pins in the distal third of the radius and two pins in the base of the second metacarpal in an open surgical manner. If acceptable alignment was achieved, percutaneous Kirschner wires (K-wires) were placed to hold the reduction (Figure 1). Then, an external fixator (Orthofix or Stryker, AO, NJ, USA, depending on the surgeon’s preference) was used to fix the fracture (Figure 2). If acceptable alignment could not be achieved, one incision was made to perform the anatomical reduction, and the fractured fragments were fixed with K-wires. A C-arm was used to check for alignment, reduction and screw placement (Figure 1).

### Open reduction and internal fixation

**Figure 3:**
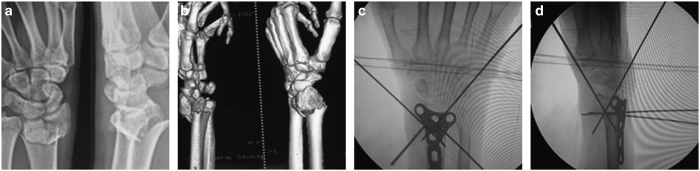
Radiographs of 73-year-old man with a comminuted intra-articular fracture of the distal radius that required treatment via Dorsal locked plating. (**a** and **b**) Anteroposterior and lateral radiographs before operation; (**c** and **d**) Anteroposterior and lateral radiographs during operation.

**Figure 4:**
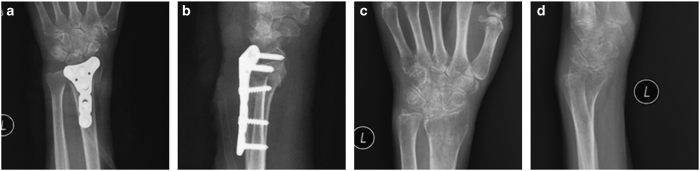
Radiographs of the man with a comminuted intra-articular fracture of the distal radius after treated by Dorsal locked plating. (**a** and **b**) Anteroposterior and lateral radiographs after one month, (**c** and **d**) Anteroposterior and lateral radiographs after one year.

A volar, dorsal, or volar/dorsal approach was used, and the plates used were 3.5 mm AO T or oblique plates. The fracture was reduced, and two pins were applied to achieve temporary fixation (Figure 3). Each fragment was identified, elevated, and reduced. Because the palmar surface of the distal radius is originally flat, application of a flat plate onto this surface was a good way to correct malrotations of the fractures in many cases (Figure 4). A C-arm was used to check for alignment, reduction and screw placement (Figure 3).

### Follow-up

Clinical follow-up was conducted at 2 and 6 weeks, and 3, 6, and 12 months postoperatively. After 1 week, patients treated via external fixation began finger exercises. Patients were seen every month for radiological follow-ups. A removable plaster cast was placed on patients in the plating group to allow free movement of their wrists and fingers.

After 3 weeks, the K-wires were removed in the outpatient clinic without anesthesia. External fixators were removed after 6 weeks. All patients had passive finger, wrist, and forearm movement. Patients underwent formal physiotherapy emphasizing activities that could prevent join stiffness and tendon adhesions. A goniometer was used to measure the range of movement of the wrist and fingers.

Two validated Swedish versions of the DASH score^14,16,17^ questionnaire were used to assess patient-rated functional results. Both questionnaires yielded a score from 0 to 100; higher scores represented greater disability. The questionnaires were completed at baseline and after 3, 6, and 12 months. Scores are presented here with the baseline scores subtracted.

After 3, 6, and 12 months, grip strength and range of movement were assessed by a physiotherapist as objective function tests. The uninjured wrists were assessed as controls. Grip strength and range of movement are expressed as percentages of those of the uninjured hand and wrist. Grip strength was adjusted by 10% for the non-dominant side. Pain was rated on a 10-point visual analog scale (0, no pain; 10, severe pain).

At each post-operative visit, radiological assessment was performed using standard anteroposterior and lateral radiographs to evaluate the union of the fracture, loss of reduction, and development of arthritis. Radial inclination, height, and tilt were measured on each radiograph (Table 1). The presence of arthritic changes was noted when observing radiographs from the post-operative visits at 6 and 12 months. All radiographs were read by a senior radiologist who had been in practice for 10 years and had extensive experience reading radiographs.

### Perceived competence

Two measures were used to investigate perceived competence. First, a Dutch version of the Self-Perception Profile was used to measure perceived competence for patients^18^ in six categories: scholastic competence, social acceptance, athletic competence, physical appearance, behavioral conduct, and global self-worth. Scores were expressed on a scale from 0 to 100. Second, a Dutch version of the Self-Perception Profile for Adolescents (CBSA) was used to measure self-esteem in patients.

### Health-related quality of life

The TNO-AZL Adult Quality of Life questionnaire (TAAQoL) was used to investigate health-related quality of life (HRQoL) at follow-up.^19^ The questionnaire contained 45 questions, divided into 12 categories. Each category consisted of 2–4 questions pertaining to gross motor functioning (4), fine motor functioning (4), pain (4), sleep (4), cognitive functioning (4), social functioning (4), daily activities (4), sexual activity (2), vitality (4), happiness (4), depressive feelings (4), and aggressiveness (3). The total score came from a simple subscale values, and higher scores demonstrated a higher quality of life.

### Statistics

The groups were compared using a *χ* ^2^-test for gender, hand dominance, fracture classification, income level, and co-morbidities, *t*-tests were used to assess differences in range of movement and radiological measurements, and a linear regression was used to analyze the follow-up DASH scores while controlling for baseline scores. The level of statistical significance was set at *P*<0.05 for each test. All statistical analyses were performed using SPSS version 17.0 software (SPSS, Chicago, IL, USA).

## Results

The groups had equal distribution in age, gender, injured side, type of work, classification of fracture, radiographic findings, and type of injury (Table 2). ALL patients had intra-articular fractures, in either the radiocarpal joint, the distal radioulnar joint, or both. The operations were performed a mean of 4.6 (1–12) days after the injury occurred. In total, 58 patients received external fixation and 65 received plating. All patients underwent follow-up for at least 1 year.

There were no open fractures in the external fixation group or the plating group. At presentation, nine patients in the external fixation group and six patients in the plating group had symptoms of carpal tunnel syndrome. The mean length of the operative procedure was 85.8 min (52–160) for the plating group and 83.6 min (50–150) for the external fixation group. Between the surgery and the subsequent 6 months, the patients in both groups attended an equal number of physiotherapy sessions.

There were no differences in the mean pain scores obtained at each interval for each group. The mean DASH scores at each follow-up point were similar for each group and were not significantly different. At each successive assessment, the mean range of wrist movement improved for each group (Table 3). There were obvious differences between the groups in pronation (*P*<0.001), supination (*P*=0.047), and extension (*P*=0.043), which reached statistical significance after 3 months. These differences were somewhat attenuated by 6 months and disappeared after 1 year. For each group, differences in the mean range of grip strength did not reach statistical significance at any point during the follow-up period.

Complication rates were not similar between the treatment methods (Table 4). There were obvious differences in rates of wound infection (*P*=0.043), pin-track infection (*P*=0.000), tendonitis (*P*=0.024), and need for further surgery, which reached statistical significance. One patient in the external fixation group and six in the plating group acquired deep wound infections. One patient in the external fixation group and seven in the plating group required further surgery because of these infections or other complications. In the plating group, one patient underwent capsulectomy and tenolysis for post-operative stiffness and one had a tendon transfer to treat rupture of the extensor pollicis longus. Ten patients had their hardware removed and required an incision and drainage with the administration of antibiotics for the treatment of a post-operative wound infection. Three patients had their hardware removed because of the symptomatic prominence of the plate and screws; two developed nonunion and required further fixation with an external fixator and bone graft, with subsequent union. For multivariate analysis, the variables that were significant in the univariate analysis were subsequently analyzed using a Cox proportional hazard model. The factor significantly related to patient cure rates was wound infection (Table 5 and Table 6).

### Perceived competence

Comparative data for perceived competence in the external fixation group and the plating group were available for 123 patients who completed the CBSA assessments after the procedure (Table 7). Most of the perceived competence scores in the external fixation group fell within the same average range as those from the plating group, except those for “athletic competence” and “close friendship”, but there were no statistically significant differences in perceived competence between the two groups.

### Health-related quality of life

Data comparing HRQoL in the external fixation group with that in the plating group were available for 123 patients who completed the TAAQoL assessment after the procedure (Table 8). The TAAQoL scores in the plating group were lower than those in the external fixation group in the “gross motor” categories (walking upstairs, bending over, and walking 500 yards; *P*=0.023; Table 8). There were no statistically significant differences in perceived competence in the other categories (Table 5 and Table 8).

## Discussion

Improved results were found with regard to range of movement in the patients undergoing plating for treatment of IFDR in the early rehabilitation period compared with those who had external fixation, but after 1 year the outcomes were similar.

Egol *et al.* ^20^ compared volar-locked plating and bridging external fixation with the use of supplementary K-wires in 280 patients and found an improved range of movement early after volar plating, but after 1 year the range of movement between the groups was similar, as were the results for grip strength and DASH scores at all time points. Marcheix *et al.* ^21^ analyzed results from 103 randomized patients with unstable extra- and intra-articular fractures treated with volar-locked plating or “mixed pinning”. The plated patients had better objective functional results and reported better DASH scores after 3 and 6 months. The 1-year results were not reported. These results are in accordance with our results, although we found better grip strength in the plating group after 6 months. Patients undergoing plate fixation had more activity than did the patients undergoing external fixation in the early stages, which may explain this difference. Our patients undergoing external fixation had somewhat better radiographic results than the patients in a study by Wilcke *et al*.^22^ This could be explained by the fact that some of our patients underwent open reduction and percutaneous K-wires were placed to hold the reduction. We found that there was not a direct relationship among the differences in grip strength and DASH score and the radiographic results because the results show that even a potentially suboptimal external fixation method produced outcomes similar to those of plating after 1 year. The minor improvements in the range of movement seen early did not lead to a better functional outcome at any stage.^20^ Compared with the external fixation group, the plating group demonstrated improved wrist movement early on, but by 1 year most of these differences were not present.

Obtaining and maintaining an acceptable reduction and allowing restoration of function are the goals of surgery for a distal radial fracture. The parameters of acceptable reduction associated with an improved outcome include restoration of radial length and minimization of the post-operative fracture gap and step. The mechanism used for fracture reduction is ligamentotaxis of the dorsal and volar capsules, which realigns the fracture with respect to the appropriate length, inclination and tilt. Our results did not show plating to be superior to external fixation in its ability to maintain proper reduction.

Higher rates of K-wire infection and hardware failure have been reported in patients treated with external fixation, and higher rates of tendon complications with internal fixation.^23^ Our patients undergoing external fixation had lower rates of deep infection and hardware failure than the patients in the study by Margaliot.^23^ We found eight pin-track infections, but most were minor and transient. One patient in the external fixation group and six in the plating group acquired deep wound infections. Using a Cox proportional hazard model, we determined that the only factor significantly related to patient cure rates was wound infection. Some studies have reported that the rate of major complications, such as redislocations requiring reoperation or complex regional pain syndrome, is higher in patients undergoing external fixation.^24^ Our external fixation group had somewhat better results than the patients in the study by Margaliot.^23^ Only three patients required further surgery for redislocation. By contrast, eight patients in the plating group required further surgery to treat wound infections and tendon-related complications.

The malunion or nonunion rates are important parameters for evaluating these two surgical methods and should be considered in the overall decision as to which method is used. In our study, only three cases in the internal fixation group and one case in the external fixation group had malunions requiring further surgery.

No psychosocial follow-up studies pertaining to IFDR treatment have been published, and our study is the first to take a long-term view (mean period of follow-up was 1 year). Furthermore, we used standardized questionnaires encompassing a wide range of psychosocial functioning domains. This method of testing health status, or HRQoL, is similar to the methodology used by McKee *et al.* ^25^ and Vitale *et al.* ^26^ Assessment of pain perception using an HRQoL questionnaire in our study yielded results similar to those of a study by McKee *et al*.^25^ In the comparison, we had expected to find differences, but the only difference we found in the TAAQoL scores between the internal fixation group and the external fixation group was a lower score in the “gross motor” category. There were no statistically significant differences in perceived competence and self-esteem in the other categories (Table 8). Our findings could be useful for deciding between external or internal procedures for treating IFDR. Patients who plan to undergo such a procedure should be informed that the procedure might involve a stressful and painful recovery. There were no statistically significant differences between the two groups with respect to recovery.

The role of this surgery in elderly patients requires further evaluation. The results of our observational study demonstrate higher complication rates in patients who underwent volar or dorsal locking plate treatment for IFDR. Furthermore, elderly patients who received greater anatomical reduction through volar or dorsal locking plate treatment did not necessarily show a better functional outcome. A large randomized, controlled trial is needed to clarify the differences in these methods and help guide treatment and counseling for elderly patients.

## Figures and Tables

**Table 1:** Mean radiological measurements in the two groups

Variables	External fixation	Plating	*P*-value
Pre-reduction			
Volar tilt/°	−14.32 (±2.33)	−14.82 (±2.56)	0.52
Radial inclination/°	13.63 (±1.3)	13.89 (±1.56)	0.67
Radial length/mm	6.83 (±2.14)	6.95 (±1.54)	0.89
			
Post-reduction			
Volar tilt/°	2.21 (±1.12)	2.31 (±1.52)	0.54
Radial inclination/°	17.76 (±2.32)	17.56 (±2.15)	0.34
radial length/mm	8.21 (±2.33)	7.76 (±2.46)	0.32
			
6 months pos operation			
Volar tilt/°	3.45 (±1.87)	3.21 (±1.77)	0.12
Radial inclination/°	19.25 (±3.97)	20.24 (±2.89)	0.56
Radial length/mm	8.87 (±3.65)	8.90 (±3.55)	0.59
			
12 months post operation			
Volar tilt/°	3.56 (±1.21)	3.31 (±1.23)	0.87
Radial inclination/°	19.32 (±3.76)	20.12 (±3.32)	0.8
Radial length/mm	9.13 (±2.13)	9.34 (±2.89)	0.78

**Table 2:** Clinical details of the patients in the two groups

Characteristics	External fixation	Plating	*P*-value
Age	67.2 (19–75)	68.7 (19–81)	0.326
Gender			0.546
Male	28	30	
Female	30	35	
Hand dominance			0.446
Right	26	41	
Left	32	24	
Medial nerve injury			0.215
Yes	9	6	
No	49	59	
Associated with ulnar fracture			0.464
Yes	5	7	
No	53	58	
Co-morbidities			
Cardiovascular disease			0.269
Yes	2	5	
No	56	60	
Endocrine disease			0.302
Yes	10	8	
No	48	57	
Pulmonary disease			0.554
Yes	2	3	
No	56	62	
Kidney disease			0.723
Yes	1	1	
No	57	64	

**Table 3:** Comparison of scores at the 3-, 6-, and 12-month follow-up in the two treatment groups

Outcome	External fixation	Percentage of uninjured side	Plating	Percentage of uninjured side	*P*-valve
Three months					
Grip strength	29.34 (±3.12)	20.89	27.54 (±2.45)	24.54	0.151
Pain/mm	1.82		2.23		0.118
Extension/%	62.87 (±4.32)	30.12	74.76 (±3.86)	23.76	0.043
Flexion/%	61.28 (±6.43)	24.76	65.87 (±4.98)	27.65	0.153
Supination/%	70.12 (±6.53)	28.65	82.76 (±4.83)	23.25	0.047
Pronation/%	81.76 (±5.87)	21.87	94.76 (±5.65)	13.29	0.001
Radial deviation/%	65.67 (±4.98)	35.65	65.54 (±7.54)	56.98	0.112
Ulnar deviation/%	65.57 (±3.76)	35.76	66.87 (±7.65)	36.85	0.142
Function (DASH score)	26.18 (±1.87)		21.98 (±3.74)		0.176
Six months					
Grip strength	51.76 (±4.21)	35.62	78.97 (±2.87)	32.17	0.021
Pain/mm	2.65 (±0.43)		2.67 (±0.87)		0.541
Extension/%	56.29 (±2.98)	14.65	57.87 (±5.87)	17.98	0.565
Flexion/%	72.13 (±3.53)	13.95	78.55 (±3.39)	15.73	0.342
Supination/%	84.21 (±3.87)	7.61	86.53 (±2.63)	8.43	0.635
Pronation/%	61.76 (±5.47)	37.85	95.12 (±5.04)	5.21	<0.001
Radial deviation/%	90.54 (±8.05)	62.15	84.89 (±7.85)	57.76	0.827
Ulnar deviation/%	75.94 (±2.97)	9.72	71.45 (±3.07)	12.23	0.397
Function (DASH score)	33.16 (±5.48)		36.65 (±7.83)		0.647
Twelve months					
Grip strength	98.07 (±8.27)	72.75	93.32 (±5.43)	68.22	0.476
Pain/mm	1.73 (±0.68)		1.65 (±0.65)		0.836
Extension/%	85.23 (±3.75)	21.76	86.48 (±5.08)	19.04	0.255
Flexion/%	81.37 (±6.02)	24.68	82.18 (±3.87)	21.98	0.632
Supination/%	98.41 (±1.64)	16.03	98.76 (±2.67)	8.98	0.627
Pronation/%	95.29 (±2.57)	5.23	99.37 (±3.59)	4.87	0.243
Radial deviation/%	99.53 (±2.76)	77.76	96.78 (±4.97)	7.38	0.347
Ulnar deviation/%	80.27 (±7.65)	23.65	79.91 (±5.81)	79.07	0.754
Function (DASH score)	18.79 (±5.54)		16.81 (±5.98)		0.276

**Table 4:** Details of the complications in both groups

Details of the complications	External fixation	Plating	*P*-value
Post-operative nerve deficit	1	3	0.367
Wound infection	1	6	0.043
Pin-track infection	8	0	0.000
Painful-retained hardware	0	1	0.343
Tendon rupture	1	2	0.627
Tendonitis	1	8	0.024
Nonunion	1	2	0.627
Further surgery	1	7	0.042

**Table 5:** Univariate analysis of risk factors relative to cure rate (logistic regression analysis)

Clinicopathological variables	Wals	*P*-value
Gender	1.376	0.775
Age	1.545	0.856
Bone loss	2.653	0.432
AO classification of fracture	3.446	0.223
Wound infection	3.216	0.043

AO, Orthopaedic Trauma Association.

**Table 6:** Multivariate analysis of risk factors relative to cure rate (logistic regression analysis)

Independent factor	*P*-value	Regression coefficient	95% confidence interval
Bone loss	0.232	0.763	0.412 (0.147-0.837)
AO classification of fracture	0.225	0.853	0.687 (0.267-0.978)
Wound infection	0.027	0.624	0.432 (0.213-0.746)

AO, Orthopaedic Trauma Association.

**Table 7:** Mean scores and standard deviation of perceived competence (ranged from 0 to 100) in the two treated groups. Higher scores indicate higher perceived competence.

Scale	External fixation	Plating	*P*-value
Scholastic competence	54±6	64±7	0.342
Social acceptance	38±5	61±4	0.263
Athletic competence	41±3	42±5	0.567
Physical appearance	51±6	56±6	0.846
Behavioral conduct	53±7	55±3	0.913
Close friendship	57±2	51±3	0.646
Global self-worth	56±7	61±4	0.265
Overall	53±4	55±5	0.734

**Table 8:** Mean HRQoL scores (TAAQoL; ranged from 0 to 100) in the two treated groups

Scale	External fixation	Plating	*P*-value
Gross motor	91±9	62±7	0.026
Fine motor	90±8	87±6	0.767
Cognitive function	98±10	96±4	0.686
Sleeping	57±5	88±7	0.045
Pain	70±6	72±7	0.445
Social function	90±6	94±8	0.654
Daily activities	82±4	82±5	0.727
Sexuality	95±7	93±7	0.887
Vitality	66±5	65±6	0.748
Happiness	75±4	73±8	0.762
Depressive feelings	83±8	97±9	0.253
Aggressiveness	85±7	82±5	0.524

HRQoL, health-related quality of life; TAAQoL, TNO-AZL Adult Quality of Life.

Higher scores indicate better quality of life.
